# Expression of X-Linked Inhibitor of Apoptosis Protein (XIAP) in Breast Cancer Is Associated with Shorter Survival and Resistance to Chemotherapy

**DOI:** 10.3390/cancers13112807

**Published:** 2021-06-04

**Authors:** Gayathri R. Devi, Pascal Finetti, Michael A. Morse, Seayoung Lee, Alexandre de Nonneville, Steven Van Laere, Jesse Troy, Joseph Geradts, Shannon McCall, Francois Bertucci

**Affiliations:** 1 Division of Surgical Sciences, Department of Surgery, Duke University Medical Center, Durham, NC 27710, USA; seayoung.lee@duke.edu; 2 Department of Pathology, Duke University Medical Center, Durham, NC 27710, USA; Shannon.mccall@duke.edu; 3 Laboratory of Predictive Oncology, Centre de Recherche en Cancérologie de Marseille (CRCM), Institut Paoli-Calmettes, INSERM UMR1068, CNRS UMR725, Aix-Marseille University, 13009 Marseille, France; FINETTIP@ipc.unicancer.fr (P.F.); denonnevillea@ipc.unicancer.fr (A.d.N.); 4 Department of Medicine, Duke University Medical Center, Durham, NC 27710, USA; michael.morse@duke.edu; 5 Department of Medical Oncology, Institut Paoli-Calmettes, 13009 Marseille, France; 6 GZA Hospitals Sint-Augustinus, 2018 Antwerp, Belgium; steven.vanlaere@gza.be; 7 Department of Biostatistics and Bioinformatics, Duke University Medical Center, Durham, NC 27710, USA; jesse.troy@duke.edu; 8 Department of Pathology and Laboratory Medicine, East Carolina University Brody School of Medicine, Greenville, NC 27858, USA; Joseph.Geradts@duke.edu

**Keywords:** anthracycline, apoptosis, cell death, HER2, luminal B, metastasis, pCR, proteomics, taxanes, TN, XIAP

## Abstract

**Simple Summary:**

The X-linked inhibitor of apoptosis protein (XIAP) is considered the most potent inhibitor of cell death, and it is well established that XIAP promotes resistance to chemotherapy, radiation, and anti-cancer immune responses. This study evaluates the correlations between XIAP expression and clinicopathological features, including disease-free survival (DFS) and pathological complete response (pCR) to chemotherapy, in more than 2300 invasive primary breast cancer samples. We found a significant association of XIAP expression with younger patients’ age (≤50 years), pathological ductal type, lower tumor grade, node-positive status, and PAM50 luminal B subtype. Analysis of molecular subtypes revealed a stronger prognostic value in HR+/HER2− tumors. Higher XIAP expression was associated with shorter DFS and lower pCR rate to chemotherapy in both uni- and multivariate analyses. All these correlations were observed at both the RNA and protein level, indicating the potential of XIAP as a promising therapeutic target in primary invasive breast cancer.

**Abstract:**

XIAP, the most potent inhibitor of cell death pathways, is linked to chemotherapy resistance and tumor aggressiveness. Currently, multiple XIAP-targeting agents are in clinical trials. However, the characterization of XIAP expression in relation to clinicopathological variables in large clinical series of breast cancer is lacking. We retrospectively analyzed non-metastatic, non-inflammatory, primary, invasive breast cancer samples for *XIAP* mRNA (*n* = 2341) and protein (*n* = 367) expression. XIAP expression was analyzed as a continuous value and correlated with clinicopathological variables. *XIAP* mRNA expression was heterogeneous across samples and significantly associated with younger patients’ age (≤50 years), pathological ductal type, lower tumor grade, node-positive status, HR+/HER2− status, and PAM50 luminal B subtype. Higher XIAP expression was associated with shorter DFS in uni- and multivariate analyses in 909 informative patients. Very similar correlations were observed at the protein level. This prognostic impact was significant in the HR+/HER2− but not in the TN subtype. Finally, *XIAP* mRNA expression was associated with lower pCR rate to anthracycline-based neoadjuvant chemotherapy in both uni- and multivariate analyses in 1203 informative patients. Higher XIAP expression in invasive breast cancer is independently associated with poorer prognosis and resistance to chemotherapy, suggesting the potential therapeutic benefit of targeting XIAP.

## 1. Introduction

Resistance to programmed cell death (apoptosis) is a hallmark of cancer, and the inhibitors of apoptosis proteins (IAP family) play a key role in regulating cell death [1,2,3]. An important member, X-linked inhibitor of apoptosis protein (XIAP), also named BIRC4 and mapped to the Xq25 chromosome region, is a multifunctional protein consisting of several domains, including three zinc-containing BIR (baculovirus IAP repeat) domains (BIR1, BIR2, and BIR3) and a C-terminal RING domain [4,5]. XIAP is considered the most potent inhibitor of apoptosis due to its ability to suppress caspase 3, 7, and 9 activation, which results in suppression of both death receptor (non-mitochondrial/extrinsic) and mitochondrial/intrinsic cell death pathways [6]. Collectively, our studies and others have shown XIAP to be involved in modulating signaling cascades of many transcription factors such as NFkB, MAPK, TGFβ, ribosomal protein S3 (RPS3), and Rho to name a few, and in turn XIAP expression and function are also regulated by these factors [2,7,8,9,10,11,12,13]. XIAP is ubiquitously expressed in almost all human tissues in the cytoplasmic compartment with a few reports identifying nuclear expression, although the associated mechanism is unclear [14]. Relevant to the mammary gland, XIAP expression is a critical factor regulating several stages of normal breast development, in particular at the end of each menstrual cycle and during involution of the mammary gland after pregnancy where XIAP levels regulate apoptosis and the reconstruction of breast ducts post lactation [15,16].

Accumulating evidence from preclinical studies strongly identifies the role of XIAP in conferring therapeutic resistance in many tumors through its inhibition of apoptosis triggered by cancer therapies [17]. In addition, XIAP can modulate downstream signaling factors involved in autophagy, necroptosis, and immunosuppression due to its ability to raise the cellular tolerance to various stress stimuli. This is largely attributed to the presence of a potent internal ribosomal entry sequence (IRES) in the long 5′ untranslated region (UTR) of *XIAP* mRNA that controls the translation of XIAP protein in response to physiological, pathological and therapeutic stress in normal and cancer cells [18]. Given that XIAP is central to apoptosis dysfunction in cancer, there is a large effort to develop anti-XIAP drugs, many of which are in clinical trials [6,19]. Indeed, association of XIAP expression with poor survival outcomes has been reported in patients with various tumors including hepatocellular cancer, gastric cancer, head and neck cancer, esophageal, and leukemia [20,21,22,23]. However, whether XIAP expression can serve as an important prognostic and/or predictive marker is not conclusive for all cancers. In breast cancer, reports suggest negative [24,25] or no correlation [26,27] between XIAP expression and survival [11,28], and to our knowledge, no study has directly investigated the correlation with response to chemotherapy in clinical breast cancer samples. Based on the strong apoptosis inhibition function of XIAP, we postulated that XIAP expression would be associated with poor survival and poor response to chemotherapy. Therefore, in this study we conducted a comprehensive analysis of XIAP gene and protein expression in a large set of non-metastatic, non-inflammatory, primary, invasive breast cancer samples to investigate XIAP expression and correlations with clinicopathological parameters including disease-free survival (DFS) and pathological complete response (pCR) to neoadjuvant chemotherapy.

## 2. Materials and Methods

### 2.1. Breast Cancer Samples and Expression Profiling

We gathered eight breast cancer gene expression data sets comprising both mRNA expression profiles generated using DNA microarrays and RNA-Seq and clinicopathological annotations. These sets were collected from the National Center for Biotechnology Information (NCBI)/Genbank GEO and TCGA databases, and authors’ website (Table S1). The final pooled data set included 2341 non-redundant non-metastatic, non-inflammatory, primary, invasive breast cancers. We also collected proteomic data from TCGA generated using Reverse Phase Protein Arrays (RPPA), which included 367 clinically annotated samples. For each of these public data sets, the patients’ consent to participate and the agreement from ethics and institutional review board had been obtained by the original authors.

### 2.2. Expression Data Analysis

Different steps of data pre-analytic processing were applied to the gene expression data as described elsewhere [29]. Briefly, it first included normalization of each data set separately using Robust Multichip Average (RMA) [30] with the non-parametric quantile algorithm to analyze the raw Affymetrix data in R (Bioconductor and associated packages employed). Then, we mapped hybridization probes across the different technological platforms as reported [31]. When the same GeneID was represented by multiple mapping probes, the probe with the highest variance in each data set was retained. The already normalized RNAseq data were log_2_-transformed. Because of known immunohistochemistry-related biases and the bimodal distribution of their respective mRNA expression level, the estrogen receptor (ER), progesterone receptor (PR), and HER2 statutes (negative/positive) were defined on mRNA expression data of *ESR1*, *PGR*, and *HER2* genes, respectively, as previously described [32]. The tumor molecular subtype was then defined as HR+/HER2− in case of ER-positivity and/or PR-positivity and HER2-negativity, HER2+ in case of HER2-positivity, and triple-negative (TN) in case of ER-negativity, PR-negativity and HER2-negativity. *XIAP* expression was measured by analyzing two probe sets with 100% identity and specificity according to the NCBI program BLASTN 2.2.29+ (Table S2). In order to avoid any arbitrary cut-off, we analyzed XIAP expression as a continuous value. Different multigene signatures were applied to each data set separately: the two major prognostic multigene classifiers of breast cancer including the Recurrence Score [33] and the 70-gene signature [34], the PAM50 molecular subtypes [35], and the genomic grade index (GGI) [36]. The normalized RPPA data did not require any pre-analytic processing.

### 2.3. Statistical Analysis

Correlations between XIAP expression (continuous value) and clinicopathological variables were analyzed using the Student’s t-test or one-way ANOVA test. Disease-free survival (DFS) was calculated for the 1–3 AJCC stages (AJCC: American Joint Commission on Cancer, 8th edition) patients from the date of diagnosis until the date of disease recurrence or death from any cause. Patients displaying a DFS event refers to patients who experienced disease recurrence or death from any cause. Follow-up was measured between the date of diagnosis and the date of last contact for event-free patients. Survival curves were generated using the Kaplan–Meier method and compared with the log-rank test. Cox regression analysis (Wald test) was done for uni- and multivariate prognostic analyses for DFS. The variables tested in univariate analyses were patients’ age at diagnosis (≤50 years vs. >50), pathological type (lobular vs. ductal vs. other), pathological axillary lymph node status (pN: negative vs. positive), pathological tumor size (pT1 vs. pT2-3), genomic grade index (GGI; low vs. high), molecular subtypes (HR+/HER2− vs. HER2+ vs. TN), the risk group defined by the two Recurrence Score and 70-gene prognostic signatures, and XIAP expression. Those variables tested in DFS analysis were assessed on the operative specimen. We also analyzed the pathological complete response (pCR) rate after anthracycline-based neoadjuvant chemotherapy, defined as absence of invasive cancer in both breast and axillary lymph nodes on the operative specimen (ypT0/Tis ypN0; AJCC 8th edition). Logistic regression was applied for uni- and multivariate analyses for pCR. Variables significant in univariate analyses (*p*-value < 0.05) were submitted to multivariate analyses. All statistical tests were two-sided at 5% level of significance. Statistical analysis used the survival package (version 2.30) in the R software (version 3.5.2; http://www.cran.r-project.org/; accessed on 20 December 2018).

## 3. Results

### 3.1. Patient Population and XIAP Expression

**Figure 1:**
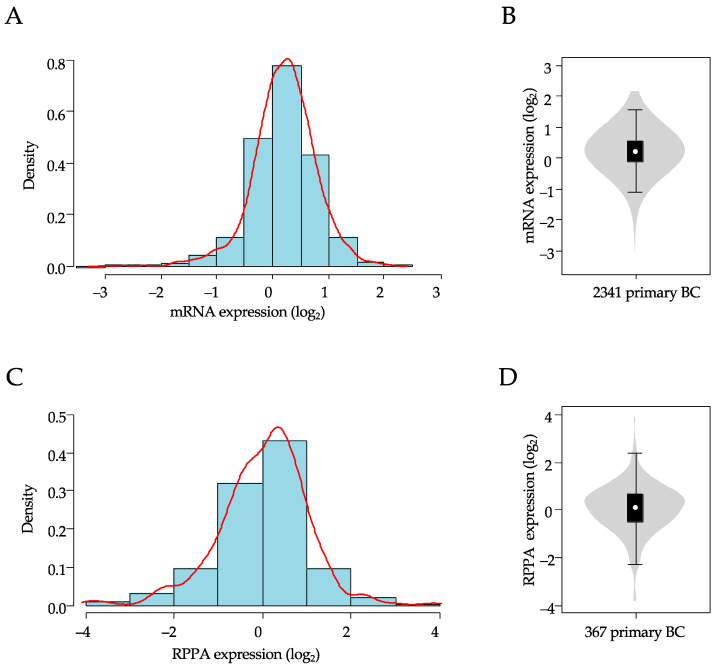
Distribution of XIAP mRNA and protein expression in breast cancer samples. (**A**) Histogram of distribution of XIAP mRNA expression levels (log2) across the 2341 clinical samples. The red line represents the density curve of distribution. (**B**) XIAP mRNA expression level (log2) reported as violin plot and box plot. (**C**) Histogram of distribution of XIAP protein expression levels (log2) across the 367 clinical samples. The red line represents the density curve of distribution. (**D**) XIAP protein expression level (log2) reported as a violin plot and box plot.

A total of 2341 breast samples were included in our analysis of *XIAP* mRNA expression. As shown in Table 1, over half of the sample size was >50-years-old at diagnosis (58%), and a higher number of tumors were ductal carcinoma (75%), high grade (68%), pT2-3 size (77%), node-positive (56%), HR+/HER2− (59%), and of luminal A+B (51%) molecular subtype. In this population, *XIAP* mRNA expression level was heterogeneous across all samples with a range of intensities over six intervals in log_2_ scale (Figure 1A,B).

Within this population, 367 TCGA samples had been profiled at the protein level using RPPA. The clinicopathological characteristics of this subset were not significantly different from those of the whole cohort. Here too, XIAP protein expression level was heterogeneous across samples with a similar range of intensities, over eight intervals in log_2_ scale (Figure 1C,D). There was a significant correlation between *XIAP* mRNA versus protein expression levels in this subset (Pearson r = 0.31, *p* = 7.92 × 10^−10^).

### 3.2. Correlations of XIAP Expression with Clinicopathological Features

Such heterogeneous expression across samples allowed a search for correlation between XIAP expression (analyzed as continuous value) and clinicopathological features. As shown in Table 1, higher *XIAP* mRNA expression was associated (*p* < 0.05; Student *t*-test or one-way ANOVA test) with patients’ age ≤50 years, pathological ductal type, lower tumor grade, node-positive status, HR+/HER2− status, and PAM50 luminal B subtype. Lower expression was found in the TN subtype. There was no correlation with pathological tumor size and HER2 status (*p* > 0.05). Mostly similar correlations were observed at the protein level (Table S3).

### 3.3. Correlations of XIAP Expression with Disease-Free Survival

**Figure 2:**
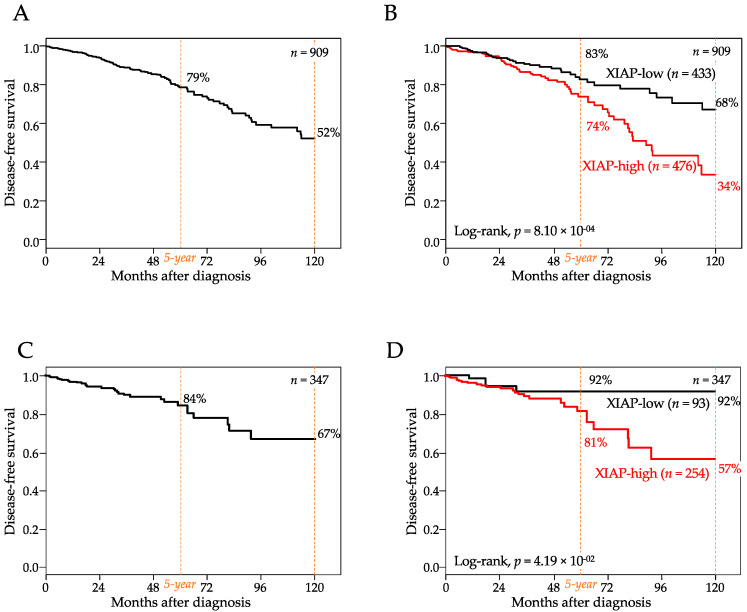
Shorter disease-free survival in high XIAP expression. (**A**) Kaplan–Meier DFS curve in 909 patients. (**B**) similar to (**A**), but according to high and low XIAP mRNA expression (the XIAP-low and XIAP-high groups were defined by the Cox model prediction). (**C**) Kaplan–Meier DFS curve in 347 patients. (**D**) similar to (**A**), but according to high and low XIAP protein expression.

Disease-free survival (DFS) data were available for 909 AJCC stage 1–3 patients, including 809 who remained disease-free and 100 who experienced an event. With a median follow-up of 24 months (range, 1–232) after diagnosis, the 5-year DFS rate was 79% (95%CI, 74–84) and the 10-year DFS rate was 52% (95%CI, 74–84; Figure 2A). In the whole population, *XIAP* mRNA expression was higher in patients who experienced a DFS event than in patients without any event (*p* = 1.55 × 10^−2^, Student *t*-test; Table 1). The “XIAP-high” group (high and low being defined by the Cox model prediction) was associated with shorter DFS (*p* = 8.1 × 10^−4^, log-rank test, Figure 2B) than the “XIAP-low” group, with respective 5-year DFS of 74% (95%CI, 67–82) and 83% (95%CI, 77–90) and respective 10-year DFS of 34% (95%CI, 21–54) and 68% (95%CI, 57–81). The hazard ratio (HR) for metastatic relapse was 1.59 (95%CI 1.17–2.15, *p* = 2.77 × 10^−3^, Wald test) in the “XIAP-high” group vs. the “XIAP-low” group (Table 2). In univariate analysis (Table 2), the other tested variables associated with shorter DFS (Wald test) included the pN-positive status (*p* = 1.40 × 10^−3^), the TN or HER2+ molecular subtypes (*p* = 7.74 × 10^−4^), and the prognostic 70-gene signature (*p* = 4.89 × 10^−3^). In multivariate analysis (Wald test; Table 2), two variables (pN and TN subtype) remained significant, whereas the 70-gene signature tended towards significance (*p* = 0.086), and the prognostic value of XIAP was maintained (HR = 1.67, 95%CI 1.20–2.31, *p* = 2.07 × 10^−3^, Wald test), suggesting independent prognostic value. The same prognostic analysis was repeated after stratification with three known prognostic factors (ER, grade, and molecular subtypes). Regarding the molecular subtypes (Figure S1A), the comparison of DFS according to XIAP expression showed significant difference in the HR+/HER2− subtype (*p* = 1.21 × 10^−4^), a difference tending towards significance in the TN subtype (*p* = 0.075), and no difference in the HER2+ subtype (*p* = 0.917). Regarding ER status (Figure S2A), the difference was significant in the ER+ tumors (*p* = 1.37 × 10^−4^), but not significant in the ER- tumors (*p* = 0.161). Finally, regarding the grade (Figure S3A), the difference was significant in the low-grade tumors (*p* = 5.25 × 10^−4^) and the high-grade tumors (*p* = 3.45 × 10^−2^). Of note, the prognostic analysis using XIAP expression as a discrete value (cut-off equal to the median expression level) gave similar results in both uni- and multivariate analyses (Table S4).

At the protein level, DFS data were available for 347 AJCC stage 1–3 patients, including 315 who remained disease-free and 32 who displayed an event. With a median follow-up of 25 months (range, 1 to 232) after diagnosis, the 5-year DFS rate was 84% (95%CI, 78–91) and the 10-year DFS rate was 67% (95%CI, 55–83; Figure 2C). Results were very similar to those observed at the transcriptional level. In the whole population, XIAP expression was higher in patients who displayed a DFS event than in patients without any event (*p* = 0.250, Student *t*-test). The “XIAP-high” group was associated with shorter DFS (*p* = 4.19 × 10^−2^, log-rank test, Figure 2D) than the “XIAP-low” group, with respective 5-year DFS of 81% (95%CI, 73–91) and 92% (95%CI, 84–100) and respective 10-year DFS of 57% (95%CI, 41–79) and 92% (95%CI, 84–100). In univariate analysis (Table S5), the other tested variables were not significantly associated with DFS (Wald test). The hazard ratio (HR) for metastatic relapse was 1.50 (95%CI 1.02–2.22, *p* = 4.15 × 10^−2^, Wald test) in the “XIAP-high” group vs. the “XIAP-low” group. The same analysis, but in each molecular subtype separately, (Figure S1B) showed a difference tending to be significant in the HR+/HER2− subtype (*p* = 0.076), non-significant difference in the TN subtype (*p* = 0.267), and no difference in the HER2+ subtype (*p* = 0.748). Analysis was repeated after stratification with ER and grade. Regarding ER status (Figure S2B). the difference tended to be significant in the ER+ tumors (*p* = 9.69 × 10^−2^), but not significant in the ER- tumors (*p* = 0.328). Regarding the grade (Figure S3B), the difference tended towards significance in the high-grade tumors (*p* = 8.37 × 10^−2^) and was not significant in the low-grade tumors (*p* = 0.216).

### 3.4. Correlations of XIAP Expression with Pathological Response to Chemotherapy

Next, we investigated the correlation between the *XIAP* mRNA expression and the pathological response to chemotherapy in the 1203 informative patients treated with anthracycline-based neoadjuvant regimen followed by surgery. The period during which the patients had been treated (available in 1108 patients) spanned, through the six informative cohorts, from 2000 to 2010. Regarding the chemotherapy regimen, 480 patients had received an anthracycline-based regimen, 668 an anthracycline/taxane-based regimen, and 55 received an anthracycline+/−taxane-based regimen. Only 10 had received trastuzumab associated to chemotherapy. Two hundred and eighty-one patients (23%) displayed pCR and 922 did not. As shown in Table 1, higher XIAP expression was observed in patients without pCR than in patients with pCR (*p* = 5.81 × 10^−4^, Student *t*-test). In univariate analysis (Table 3), the XIAP status was associated with pCR, as were other variables including the tumor grade and the molecular subtypes with higher pCR rate for GGI high versus low (OR = 2.10, 95%CI 1.70–2.80; *p* = 7.65 × 10^−7^), for HER2+ versus HR+/HER2− (OR = 3.80, 95%CI 2.70–5.40; *p* = 1.12 × 10^−10^) and for TN versus HR+/HER2− (OR = 3.60, 95%CI 2.80–4.70; *p* = 2.22 × 10^−15^). Here too, in multivariate analysis (Table 3), XIAP expression remained significantly associated with lower pCR rate (*p* = 1.28 × 10^−2^, logit function). Similar results were observed by using XIAP expression as a discrete value (Table S6).

## 4. Discussion

Although there have been significant improvements in understanding breast cancer biology, therapeutic approaches are largely dependent on and guided by molecular profiling that categorizes the tumor based on the three receptors/biomarkers: ER, PR and HER2 [37]. Unfortunately, recent emerging global trends are showing increased breast cancer mortality [38] attributed to treatment resistance and highly proliferative breast cancer variants within these subtypes [39,40]. Thus, the development of new therapies that address resistance and proliferative mechanisms is crucial.

Programmed cell death/apoptosis is vital for homeostasis and regulation of cell survival [41]. It is commonly dysregulated in many cancers and during adaptation to therapeutic stress leading to clonal expansion of aggressive, proliferative tumor cells, which exhibit resistance to hypoxia, radiation, chemotherapy, and other survival pressures [42,43]. Among the two families of known apoptosis regulators (IAP and Bcl-2 families of proteins), XIAP is considered the most potent inhibitor of cell death and an attractive therapeutic target due to its ability to suppress caspase activation via both intrinsic and extrinsic pathways and its ability to act as a signaling intermediate in tumor cell survival, immune and inflammatory pathways [17,44]. Based on strong evidence that XIAP expression in cancer cells promotes resistance to chemotherapy and radiation as well as elicit anti-cancer immune responses, the past decade has seen development of XIAP-specific targeting using RNA approaches [45,46,47,48,49], pan-IAP peptidomimetics, and small molecule inhibitors [17,19,50,51,52,53]. In particular, Smac mimetics have been instrumental in revealing the role for IAPs in regulating TNF receptor signaling [54] and have shown promising results in sensitizing cancer cell lines to conventional chemotherapies by occupying the BIR domains that normally interact with caspases [55]. Various Smac mimetics are in early phase clinical trials, such as Birinapant in phase II clinical trial for the treatment of solid tumors in combination with Pembrolizumab [56,57,58]. However, characterization of XIAP expression in relation to both clinicopathological markers and clinical outcomes using larger series of well annotated samples of invasive breast cancers is lacking. To our knowledge, the present study is the largest clinical series of primary invasive breast cancers analyzed for XIAP expression.

We found a significant association between higher XIAP expression (mRNA and protein datasets) and several prognostic clinicopathological variables. Curiously, the latter included both poor-prognosis factors (younger age, ductal type, PAM50 luminal B subtype, and pathological node-positive status), and good-prognosis factors (lower tumor grade, ER+ status, PR+ status, HR+/HER2− status, and PAM50 luminal A subtype). Such heterogeneity in correlation with prognostic factors is difficult to explain. Similar observations were reported in one study of 92 patients with correlation of higher IAP protein expression with both a poor-prognosis variable (higher tumor size pT2-3) and a good-prognosis variable (ER+ status) [26]. Three other published studies reported different and contradictory results: high XIAP protein expression was associated with no variable tested in one study [24], with HER2+ status and *TP53* mutations in the second one [27], and with higher grade, tumor size pT2-3, ductal type, and TN status in the third one [25]. For comparison, the literature data are more consistent and coherent for Bcl-2, another major inhibitor of apoptosis, whose expression is associated with good-prognosis factors such as ER+, low grade, and smaller tumor size [59].

Most importantly, patients with higher mRNA levels of XIAP in their tumors exhibited shorter 5-year and 10-year DFS than those with lower levels of XIAP, and these associations were also found at the protein level. Thus, in both cohorts (RNA and protein/RPPA cohorts), XIAP expression was a poor-prognosis factor. Interestingly, this result was observed in analyses of XIAP expression as a continuous value, which showed a linear relation between the risk of event and expression level, and as discrete value (cut-off equal to median expression level), which showed higher risk of event in “XIAP-high” versus “XIAP-low” samples. This poor-prognosis value of high XIAP expression may appear as paradoxical given its association with good-prognosis variables (HR+/HER2−, ER+, low grade). However, high expression was not only associated with poor-prognosis variables in the whole series, but also with shorter DFS in HR+/HER2− tumors and TN tumors (Figure S1), in ER+ tumors and ER- tumors (Figure S2), and in low-grade tumors and high-grade tumors (Figure S3). Indeed, the prognostic value was maintained when subjected to multivariate analysis to the classical prognostic variables including the 70-gene signature [34,60], suggesting that apoptosis (reflected by XIAP expression) and tumor cell proliferation (reflected by the 70-gene signature) provide complementary prognostic information. In the literature, the unfavorable prognostic value of high XIAP expression has been reported as significant in multivariate analysis for overall survival in two studies [24,25], as tending towards significance for PFS in one study [26], and as absent in another one [27].

We also report in a uni- and multivariate analysis of more than 1200 patients that higher XIAP tumor expression was associated with lower pCR rate to anthracycline-based neoadjuvant chemotherapy. Here too, this predictive value, independent from the genomic grade (GGI, mostly related to cell proliferation), reveals the predictive complementarity between apoptosis and proliferation for the therapeutic response to chemotherapy. Further, as pCR is associated with favorable disease-free and overall survival in early-stage breast cancer, limiting the negative impact of XIAP during neoadjuvant therapy may result in important gains in efficacy. This correlation of XIAP expression with poorer response to chemotherapy, as well as with shorter DFS, is in agreement with the role of apoptosis inhibition in cancer formation and progression [3]. This is supported by prior studies reporting the expression of another key anti-apoptotic protein Bcl-2 to be associated with lesser chemosensitivity [61]. Paradoxically, though Bcl-2 expression is associated with longer survival in breast cancer [59,62] and unlike XIAP that suppresses both death receptor/extrinsic and mitochondrial apoptosis, Bcl-2 can only inhibit intrinsic/mitochondrial-mediated apoptosis, suggesting that targeting XIAP may be more relevant to treatment of breast cancer.

## 5. Conclusions

Our study supports that, at both the transcriptional and translational levels, higher XIAP expression in primary invasive breast cancer is associated with poorer prognosis and resistance to chemotherapy. The strengths of our study include the size of the series, which to our knowledge represents the largest prognostic/predictive study of XIAP expression reported so far in breast cancer, the analysis per molecular subtype, the independent prognostic and predictive values, and analysis at both mRNA and protein levels. The main limitation is the retrospective nature of our series and associated biases, warranting further validation in larger retrospective and prospective clinical series before being able to recognize XIAP expression as a validated biomarker useful in clinical practice. Furthermore, given this potential role of XIAP and apoptosis regarding the clinical outcome of breast cancer patients, the manipulation of XIAP with XIAP/Smac mimetic therapies under development might provide new therapeutic weapons for treating these poor-prognosis tumors, and functional studies with pre-clinical models are warranted.

## Figures and Tables

**Table 1:** XIAP mRNA expression and clinicopathological variables.

Variables		*n*	Global	XIAP mRNA	
			*n* (%)	Mean (Range)	*p*-Value *
Age at diagnosis (year)					3.81 × 10^−2^
	≤50	991	991 (42%)	0.23 (−2.3–2.0)	
	>50	1343	1343 (58%)	0.17 (−3.0–2.1)	
Pathological type					4.35 × 10^−2^
	IDC	1211	1211 (75%)	0.19 (−3.0–2.1)	
	ILC	215	215 (13%)	0.12 (−2.4–1.3)	
	other	190	190 (12%)	0.17 (−2.6–2.0)	
Pathological lymph node (pN)					9.21 × 10^−2^
	negative	517	517 (44%)	0.06 (−3.0–2.0)	
	positive	670	670 (56%)	0.17 (−2.1–1.9)	
Pathological size (pT)					0.2
	pT1	321	321 (23%)	0.21 (−2.4–1.93)	
	pT2-3	1070	1070 (77%)	0.12 (−3.0–2.0)	
Genomic grade (GGI)					1.15 × 10^−3^
	low	758	758 (32%)	0.24 (−2.4–2.0)	
	high	1583	1583 (68%)	0.18 (−3.0–2.1)	
ER status **					1.73 × 10^−12^
	negative	917	917 (39%)	0.1 (−2.6–2.1)	
	positive	1424	1424 (61%)	0.26 (−3.0–2.0)	
PR status **					4.68 × 10^−7^
	negative	1419	1419 (61%)	0.15 (−3.0–2.1)	
	positive	922	922 (39%)	0.27 (−2.7–1.9)	
HER2 status **					0.355
	negative	2068	2068 (88%)	0.2 (−3.0–2.1)	
	positive	273	273 (12%)	0.17 (−1.6–1.8)	
Molecular subtype mRNA status					2.86 × 10^−14^
	HR+/HER2−	1382	1382 (59%)	0.26 (−3.0–2.0)	
	HER2+	273	273 (12%)	0.17 (−1.6–1.8)	
	TN	686	686 (29%)	0.07 (−2.6–2.1)	
PAM50 subtypes					6.93 × 10^−23^
	basal	641	641 (27%)	0.06 (−2.6–2.1)	
	HER2	320	320 (14%)	0.12 (−3.0–1.9)	
	luminal A	668	668 (29%)	0.27 (−2.4–2.0)	
	luminal B	516	516 (22%)	0.35 (−1.8–2.0)	
	normal-like	196	196 (8%)	0.14 (−1.7–1.9)	
DFS event	no	809	809 (89%)	0.13 (−3.0–2.0)	1.55 × 10^−2^
	yes	100	100 (11%)	0.33 (−1.8–1.5)	
5-year DFS [95% CI]		909	79% [74–84]		
Pathological complete response (pCR)					5.81 × 10^−4^
	no	922	922 (77%)	0.26 (−1.4–2.1)	
	yes	281	281 (23%)	0.15 (−2.3–1.9)	

GGI, genomic grade index; IDC, invasive ductal carcinoma; ILC, invasive lobular carcinoma. * Student’s *t*-test or one-way ANOVA; **, mRNA status.

**Table 2:** Univariate and multivariate analyses for DFS in the “RNA population”.

Variables	Univariate			Multivariate		
	*n*	HR [95%CI]	*p*-Value	*n*	HR [95%CI]	*p*-Value
Age at diagnosis, >50 vs. ≤50 years	909	1.24 [0.81–1.88]	0.323			
Genomic grade (GGI), high vs. low	909	1.30 [0.81–2.07]	0.275			
Pathological lymph node, pos. vs. neg.	776	2.05 [1.32–3.18]	1.40 × 10^−3^	776	1.94 [1.23–3.05]	4.03 × 10^−3^
Pathological size, pT2-3 vs. pT1	908	1.15 [0.74–1.79]	0.536			
Pathological type, ILC vs. IDC	909	0.54 [0.28–1.04]	0.182			
Pathological type, other vs. IDC		0.99 [0.55–1.81]				
Mol. subtype, HER2+ vs. HR+/HER2−	909	2.18 [1.31–3.63]	7.72 × 10^−4^	776	1.70 [0.95–3.04]	0.073
Mol. subtype, TN vs. HR+/HER2−		2.13 [1.32–3.43]		776	2.57 [1.52–4.35]	4.35 × 10^−4^
Amsterdam 70-gene risk, high vs. low	909	2.46 [1.31–4.60]	4.89 × 10^−3^	776	1.77 [0.92-3.41]	0.086
Recurrence Score risk, high vs. low	909	1.60 [0.98–2.60]	0.168			
Recurrence Score risk, intermediate vs. low		1.33 [0.70–2.51]				
*XIAP* continuous expression	909	1.59 [1.17–2.15]	2.77 × 10^−3^	776	1.67 [1.20–2.31]	2.07 × 10^−3^

GGI, genomic grade index; IDC, invasive ductal carcinoma; ILC, invasive lobular carcinoma; HR, hazards ratio.

**Table 3:** Univariate and multivariate analyses for pCR to neoadjuvant chemotherapy.

Variables	Univariate			Multivariate		
	*n*	OR [CI95]	*p*-Value	*n*	OR [CI95]	*p*-Value
Age at diagnosis, >50 vs. ≤50 years	1202	0.86 [0.68–1.10]	0.262			
Genomic grade (GGI), high vs. low	1203	2.10 [1.70–2.80]	7.65 × 10^−7^	1203	1.60 [1.20–2.10]	2.72 × 10^−3^
Pathological type, ILC vs. IDC	510	1.60 [0.63–4.30]	0.397			
Pathological type, other vs. IDC	510	0.75 [0.46–1.20]	0.314			
Mol. subtype, HER2+ vs. HR+/HER2−	1203	3.80 [2.70–5.40]	1.12 × 10^−10^	1203	3.40 [2.40–4.80]	7.85 × 10^−9^
Mol. subtype, TN vs. HR+/HER2−	1203	3.60 [2.80–4.70]	2.22 × 10^−15^	1203	3.00 [2.30–4.00]	3.96 × 10^−11^
*XIAP* continuous expression	1203	0.59 [0.46–0.76]	5.12 × 10^−4^	1203	0.67 [0.52–0.87]	1.28 × 10^−2^

GGI, genomic grade index; IDC, invasive ductal carcinoma; ILC, invasive lobular carcinoma; OR, Odds-ratio.

## Data Availability

All data sets of primary breast cancer were downloaded from the Gene Expression Omnibus (GEO, https://www.ncbi.nlm.nih.gov/geo/), ArrayExpress (https://www.ebi.ac.uk/arrayexpress/), Genomic Data Commons (GDC, https://portal.gdc.cancer.gov/) and cBioPortal (https://www.cbioportal.org/) databases. The data references and accessed date are described in Table S1.

## Supplementary Materials

The following are available online at https://www.mdpi.com/article/10.3390/cancers13112807/s1, Figure S1: Disease-free survival according to XIAP expression in each molecular subtype, Figure S2: Disease-free survival according to XIAP expression in each ER status-based class, Figure S3: Disease-free survival according to XIAP expression in each grade-based class, Table S1: List of breast cancer mRNA data sets included in the study, Table S2: List of XIAP probe sets analyzed; Table S3: XIAP protein expression and clinicopathological variables, Table S4: Univariate and multivariate analyses for DFS in the “RNA population” by using XIAP expression as discrete value. Table S5: Univariate and multivariate analyses for DFS in the “RPPA population”. Table S6: Univariate and multivariate analyses for pCR to neoadjuvant chemotherapy by using XIAP expression as discrete value. References [63,64,65,66,67,68,69] are referred to in Supplementary Materials.

## References

[1] Lewis J., Burstein E., Reffey S.B., Bratton S.B., Roberts A.B., Duckett C.S. (2004). Uncoupling of the signaling and caspase-inhibitory properties of X-linked inhibitor of apoptosis. J. Biol. Chem..

[2] Gyrd-Hansen M., Meier P. (2010). IAPs: From caspase inhibitors to modulators of NF-κB, inflammation and cancer. Nat. Rev. Cancer.

[3] Hanahan D., Weinberg R.A. (2000). The Hallmarks of Cancer. Cell.

[4] Jost P.J., Vucic D. (2020). Regulation of Cell Death and Immunity by XIAP. Cold Spring Harb. Perspect. Biol..

[5] Harlin H., Reffey S.B., Duckett C.S., Lindsten T., Thompson C.B. (2001). Characterization of XIAP-deficient mice. Mol. Cell. Biol..

[6] Tu H., Costa M. (2020). XIAP’s Profile in human cancer. Biomolecules.

[7] Evans M.K., Sauer S.J., Nath S., Robinson T.J., Morse M.A., Devi G.R. (2016). X-linked inhibitor of apoptosis protein mediates tumor cell resistance to antibody-dependent cellular cytotoxicity. Cell Death Dis..

[8] Allensworth J.L., Aird K.M., Aldrich A.J., Batinic-Haberle I., Devi G.R. (2012). XIAP inhibition and generation of reactive oxygen species enhances TRAIL sensitivity in inflammatory breast cancer cells. Mol. Cancer Ther..

[9] Huang X., Wu Z., Mei Y., Wu M. (2013). XIAP inhibits autophagy via XIAP-Mdm2-p53 signalling. EMBO J..

[10] Lalaoui N., Vaux D.L. (2018). Recent advances in understanding inhibitor of apoptosis proteins. F1000Research.

[11] Evans M.K., Brown M.C., Geradts J., Bao X., Robinson T.J., Jolly M.K., Vermeulen P.B., Palmer G.M., Gromeier M., Levine H. (2018). XIAP regulation by MNK links MAPK and NFκB signaling to determine an aggressive breast cancer phenotype. Cancer Res..

[12] Ono H., Iizumi Y., Goi W., Sowa Y., Taguchi T., Sakai T. (2017). Ribosomal protein S3 regulates XIAP expression independently of the NF-κB pathway in breast cancer cells. Oncol. Rep..

[13] Yu Y., Jin H., Xu J., Gu J., Li X., Xie Q., Huang H., Li J., Tian Z., Jiang G. (2018). XIAP overexpression promotes bladder cancer invasion in vitro and lung metastasis in vivo via enhancing nucleolin-mediated Rho-GDIβ mRNA stability. Int. J. Cancer.

[14] Liston P., Young S.S., Mackenzie A.E., Korneluk R.G. (1997). Life and death decisions: The role of the IAPs in modulating programmed cell death. Apoptosis.

[15] Owens T.W., Foster F.M., Tanianis-Hughes J., Cheung J.Y., Brackenbury L., Streuli C.H. (2010). Analysis of inhibitor of apoptosis protein family expression during mammary gland development. BMC Dev. Biol..

[16] Yang L.L., Piccart M.J., Hung M.-C., Solin L.J., Cardoso F., Wood W.C. (2006). Mechanisms of apoptosis resistance in breast cancer. Breast Cancer and Molecular Medicine.

[17] Abbas R., Larisch S. (2020). Targeting XIAP for promoting cancer cell death-the story of ARTS and SMAC. Cells.

[18] Holcik M., Lefebvre C., Yeh C., Chow T., Korneluk R.G. (1999). A new internal-ribosome-entry-site motif potentiates XIAP-mediated cytoprotection. Nat. Cell Biol..

[19] Shahar N., Larisch S. (2020). Inhibiting the inhibitors: Targeting anti-apoptotic proteins in cancer and therapy resistance. Drug Resist. Updates.

[20] Tamm I., Kornblau S.M., Segall H., Krajewski S., Welsh K., Kitada S., Scudiero D.A., Tudor G., Qui Y.H., Monks A. (2000). Expression and prognostic significance of IAP-family genes in human cancers and myeloid leukemias. Clin. Cancer Res..

[21] Shi Y.-H., Ding W.-X., Zhou J., He J.-Y., Xu Y., Gambotto A.A., Rabinowich H., Fan J., Yin X.-M. (2008). Expression of X-linked inhibitor-of-apoptosis protein in hepatocellular carcinoma promotes metastasis and tumor recurrence. Hepatology.

[22] Dizdar L., Jünemann L.M., Werner T.A., Verde P.E., Baldus S.E., Stoecklein N.H., Knoefel W.T., Krieg A. (2018). Clinicopathological and functional implications of the inhibitor of apoptosis proteins survivin and XIAP in esophageal cancer. Oncol. Lett..

[23] Gao X., Zhang L., Wei Y., Yang Y., Li J., Wu H., Yin Y. (2019). Prognostic value of XIAP level in patients with various cancers: A systematic review and meta-analysis. J. Cancer.

[24] Zhang Y., Zhu J., Tang Y., Li F., Zhou H., Peng B., Zhou C., Fu R. (2011). X-linked inhibitor of apoptosis positive nuclear labeling: A new independent prognostic biomarker of breast invasive ductal carcinoma. Diagn. Pathol..

[25] Hussain A.R., Siraj A.K., Ahmed M., Bu R., Pratheeshkumar P., Alrashed A.M., Qadri Z., Ajarim D., Al-Dayel F., Beg S. (2017). XIAP over-expression is an independent poor prognostic marker in Middle Eastern breast cancer and can be targeted to induce efficient apoptosis. BMC Cancer.

[26] Pluta P., Jeziorski A., Cebula-Obrzut A.P., Wierzbowska A., Piekarski J., Smolewski P. (2015). Expression of IAP family proteins and its clinical importance in breast cancer patients. Neoplasma.

[27] Xu Y.C., Liu Q., Dai J.Q., Yin Z.Q., Tang L., Ma Y., Lin X.L., Wang H.X. (2014). Tissue microarray analysis of X-linked inhibitor of apoptosis (XIAP) expression in breast cancer patients. Med. Oncol..

[28] Arora J., Sauer S.J., Tarpley M., Vermeulen P., Rypens C., Van Laere S., Williams K.P., Devi G.R., Dewhirst M.W. (2017). Inflammatory breast cancer tumor emboli express high levels of anti-apoptotic proteins: Use of a quantitative high content and high-throughput 3D IBC spheroid assay to identify targeting strategies. Oncotarget.

[29] Bertucci F., Finetti P., Simeone I., Hendrickx W., Wang E., Marincola F.M., Viens P., Mamessier E., Ceccarelli M., Birnbaum D. (2018). The immunologic constant of rejection classification refines the prognostic value of conventional prognostic signatures in breast cancer. Br. J. Cancer.

[30] Irizarry R.A., Hobbs B., Collin F., Beazer-Barclay Y.D., Antonellis K.J., Scherf U., Speed T.P. (2003). Exploration, normalization, and summaries of high density oligonucleotide array probe level data. Biostatistics.

[31] Bertucci F., Finetti P., Viens P., Birnbaum D. (2014). EndoPredict predicts for the response to neoadjuvant chemotherapy in ER-positive, HER2-negative breast cancer. Cancer Lett..

[32] Lehmann B.D., Bauer J.A., Chen X., Sanders M.E., Chakravarthy A.B., Shyr Y., Pietenpol J.A. (2011). Identification of human triple-negative breast cancer subtypes and preclinical models for selection of targeted therapies. J. Clin. Investig..

[33] Paik S., Shak S., Tang G., Kim C., Baker J., Cronin M., Baehner F.L., Walker M.G., Watson D., Park T. (2004). A multigene assay to predict recurrence of tamoxifen-treated, node-negative breast cancer. N. Engl. J. Med..

[34] Van De Vijver M.J., He Y.D., Van’t Veer L.J., Dai H., Hart A.A., Voskuil D.W., Schreiber G.J., Peterse J.L., Roberts C., Marton M.J. (2002). A gene-expression signature as a predictor of survival in breast cancer. N. Engl. J. Med..

[35] Parker J.S., Mullins M., Cheang M.C., Leung S., Voduc D., Vickery T., Davies S., Fauron C., He X., Hu Z. (2009). Supervised risk predictor of breast cancer based on intrinsic subtypes. J. Clin. Oncol..

[36] Sotiriou C., Wirapati P., Loi S., Harris A., Fox S., Smeds J., Nordgren H., Farmer P., Praz V., Haibe-Kains B. (2006). Gene expression profiling in breast cancer: Understanding the molecular basis of histologic grade to improve prognosis. J. Natl. Cancer Inst..

[37] Koboldt D.C., Fulton R.S., McLellan M.D., Schmidt H., Kalicki-Veizer J., McMichael J.F., Fulton L.L., Dooling D.J., Ding L., Mardis E.R. (2012). Comprehensive molecular portraits of human breast tumours. Nature.

[38] Heer E., Harper A., Escandor N., Sung H., McCormack V., Fidler-Benaoudia M.M. (2020). Global burden and trends in premenopausal and postmenopausal breast cancer: A population-based study. Lancet Glob. Health.

[39] U.S. Breast Cancer Statistics. https://www.breastcancer.org/symptoms/understand_bc/statistics.

[40] Redig A.J., McAllister S.S. (2013). Breast cancer as a systemic disease: A view of metastasis. J. Intern. Med..

[41] Fulda S., Gorman A.M., Hori O., Samali A. (2010). Cellular stress responses: Cell survival and cell death. Int. J. Cell. Biol..

[42] Reed J.C. (1999). Dysregulation of apoptosis in cancer. J. Clin. Oncol..

[43] Pazarentzos E., Bivona T.G. (2015). Adaptive stress signaling in targeted cancer therapy resistance. Oncogene.

[44] Michie J., Kearney C.J., Hawkins E.D., Silke J., Oliaro J. (2020). The immuno-modulatory effects of inhibitor of apoptosis protein antagonists in cancer immunotherapy. Cells.

[45] Amantana A., London C.A., Iversen P.L., Devi G.R. (2004). X-linked inhibitor of apoptosis protein inhibition induces apoptosis and enhances chemotherapy sensitivity in human prostate cancer cells. Mol. Cancer Ther..

[46] LaCasse E.C. (2013). Pulling the plug on a cancer cell by eliminating XIAP with AEG35156. Cancer Let..

[47] Cao C., Mu Y., Hallahan D.E., Lu B. (2004). XIAP and survivin as therapeutic targets for radiation sensitization in preclinical models of lung cancer. Oncogene.

[48] Devi G.R., Beer T.M., Corless C.L., Arora V., Weller D.L., Iversen P.L. (2005). In vivo bioavailability and pharmacokinetics of a c-MYC antisense phosphorodiamidate morpholino oligomer, AVI-4126, in solid tumors. Clin. Cancer Res..

[49] Arora V., Devi G.R., Iversen P.L. (2004). Neutrally charged phosphorodiamidate morpholino antisense oligomers: Uptake, efficacy and pharmacokinetics. Curr. Pharm. Biotechnol..

[50] Panayotopoulou E.G., Müller A.-K., Börries M., Busch H., Hu G., Lev S. (2017). Targeting of apoptotic pathways by SMAC or BH3 mimetics distinctly sensitizes paclitaxel-resistant triple negative breast cancer cells. Oncotarget.

[51] Cong H., Xu L., Wu Y., Qu Z., Bian T., Zhang W., Xing C., Zhuang C. (2019). Inhibitor of apoptosis protein (IAP) antagonists in anticancer agent discovery: Current status and perspectives. J. Med. Chem..

[52] Xie X., Lee J., Liu H., Pearson T., Lu A.Y., Tripathy D., Devi G.R., Bartholomeusz C., Ueno N.T. (2021). Birinapant enhances gemcitabine’s antitumor efficacy in triple-negative breast cancer by inducing intrinsic pathway–dependent apoptosis. Mol. Cancer Ther..

[53] Allensworth J.L., Sauer S.J., Lyerly H.K., Morse M.A., Devi G.R. (2013). Smac mimetic Birinapant induces apoptosis and enhances TRAIL potency in inflammatory breast cancer cells in an IAP-dependent and TNF-alpha-independent mechanism. Breast Cancer Res. Treat..

[54] Dougan S.K., Dougan M. (2018). Regulation of innate and adaptive antitumor immunity by IAP antagonists. Immunotherapy.

[55] Fulda S. (2015). Promises and challenges of smac mimetics as cancer therapeutics. Clin. Cancer Res..

[56] Amaravadi R.K., Senzer N.N., Martin L.P., Schilder R.J., LoRusso P., Papadopoulos K.P., Weng D.E., Graham M., Adjei A.A. (2013). A phase I study of birinapant (TL32711) combined with multiple chemotherapies evaluating tolerability and clinical activity for solid tumor patients. J. Clin. Oncol..

[57] Amaravadi R.K., Schilder R.J., Martin L.P., Levin M., Graham M.A., Weng D.E., Adjei A.A. (2015). A phase I study of the SMAC-mimetic birinapant in adults with refractory solid tumors or lymphoma. Mol. Cancer Ther..

[58] Lalaoui N., Merino D., Giner G., Vaillant F., Chau D., Liu L., Kratina T., Pal B., Whittle J.R., Etemadi N. (2020). Targeting triple-negative breast cancers with the Smac-mimetic birinapant. Cell. Death Differ..

[59] Callagy G.M., Pharoah P.D., Pinder S.E., Hsu F.D., Nielsen T.O., Ragaz J., Ellis I.O., Huntsman D., Caldas C. (2006). Bcl-2 is a prognostic marker in breast cancer independently of the nottingham prognostic index. Clin. Cancer Res..

[60] Van ‘t Veer L.J., Dai H., van de Vijver M.J., He Y.D., Hart A.A.M., Mao M., Peterse H.L., van der Kooy K., Marton M.J., Witteveen A.T. (2002). Gene expression profiling predicts clinical outcome of breast cancer. Nature.

[61] Yang D., Chen M.B., Wang L.Q., Yang L., Liu C.Y., Lu P.H. (2013). Bcl-2 expression predicts sensitivity to chemotherapy in breast cancer: A systematic review and meta-analysis. J. Exp. Clin. Cancer Res..

[62] Callagy G.M., Webber M.J., Pharoah P.D.P., Caldas C. (2008). Meta-analysis confirms BCL2 is an independent prognostic marker in breast cancer. BMC Cancer.

[63] Hess K.R., Anderson K., Symmans W.F., Valero V., Ibrahim N., Mejia J.A., Booser D., Theriault R.L., Buzdar A.U., Dempsey P.J. (2006). Pharmacogenomic Predictor of Sensitivity to Preoperative Chemotherapy with Paclitaxel and Fluorouracil, Doxorubicin, and Cyclophosphamide in Breast Cancer. J. Clin. Oncol..

[64] Bonnefoi H., Potti A., Delorenzi M., Mauriac L., Campone M., Tubiana-Hulin M., Petit T., Rouanet P., Jassem J., Blot E. (2007). Validation of Gene Signatures That Predict the Response of Breast Cancer to Neoadjuvant Chemotherapy: A Substudy of the EORTC 10994/BIG 00-01 Clinical Trial. Lancet Oncol..

[65] Iwamoto T., Bianchini G., Booser D., Qi Y., Coutant C., Shiang C.Y.-H., Santarpia L., Matsuoka J., Hortobagyi G.N., Symmans W.F. (2011). Gene Pathways Associated with Prognosis and Chemotherapy Sensitivity in Molecular Subtypes of Breast Cancer. J. Natl. Cancer Inst..

[66] Tabchy A., Valero V., Vidaurre T., Lluch A., Gomez H., Martin M., Qi Y., Barajas-Figueroa L.J., Souchon E., Coutant C. (2010). Evaluation of a 30-Gene Paclitaxel, Fluorouracil, Doxorubicin, and Cyclophosphamide Chemotherapy Response Predictor in a Multicenter Randomized Trial in Breast Cancer. Clin. Cancer Res..

[67] Desmedt C., Di Leo A., de Azambuja E., Larsimont D., Haibe-Kains B., Selleslags J., Delaloge S., Duhem C., Kains J.-P., Carly B. (2011). Multifactorial Approach to Predicting Resistance to Anthracyclines. J. Clin. Oncol..

[68] Hatzis C., Pusztai L., Valero V., Booser D.J., Esserman L., Lluch A., Vidaurre T., Holmes F., Souchon E., Wang H. (2011). A Genomic Predictor of Response and Survival Following Taxane-Anthracycline Chemotherapy for Invasive Breast Cancer. JAMA.

[69] Popovici V., Chen W., Gallas B.G., Hatzis C., Shi W., Samuelson F.W., Nikolsky Y., Tsyganova M., Ishkin A., Nikolskaya T. (2010). Effect of Training-Sample Size and Classification Difficulty on the Accuracy of Genomic Predictors. Breast Cancer Res..

