# Supplementary material for: Expression of X-Linked Inhibitor of Apoptosis Protein (XIAP) in Breast Cancer Is Associated with Shorter Survival and Resistance to Chemotherapy
Source: Cancers (Basel). 2021 Jun 4;13(11):2807. doi: 10.3390/cancers13112807 (PMC8200223; doi:10.3390/cancers13112807)
Supplement: Supplementary file 1 [file cancers-13-02807-s001.zip › cancers-1205840-supplementary.pdf]

Supplementary Materials

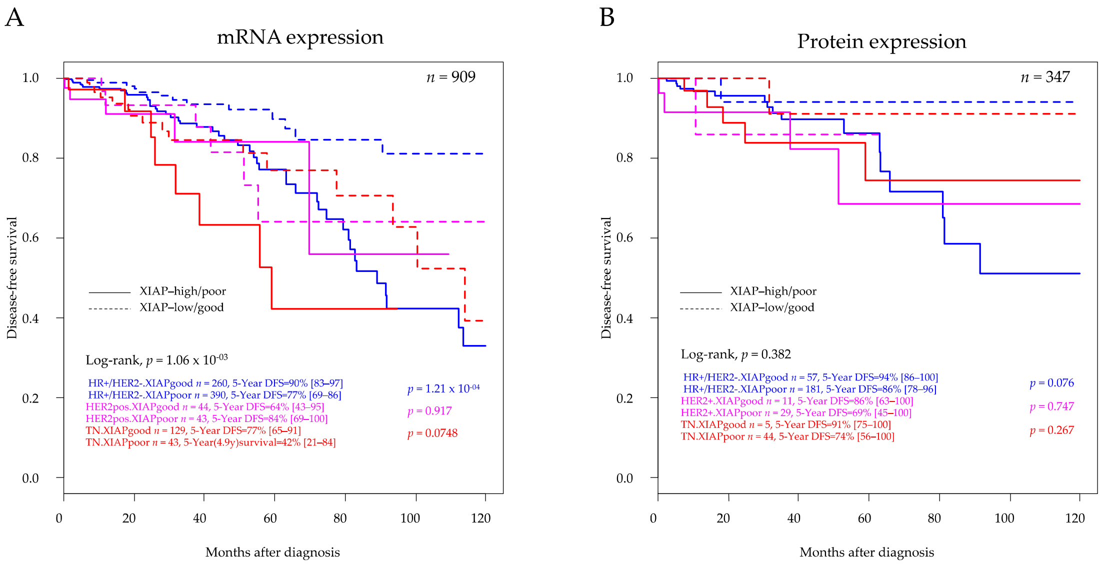

Figure S1. Disease-free survival according to XIAP expression in each molecular subtype. (**A**) Kaplan-Meier DFS curve in 909 patients *per* molecular subtype and according to high and low *XIAP* mRNA expression. The XIAP-low and XIAP-high groups were defined by the Cox model prediction. (**B**) Kaplan-Meier DFS curve in 347 patients *per* molecular subtype and according to high and low XIAP protein expression. The XIAP-low and XIAP-high groups were defined by the Cox model prediction.

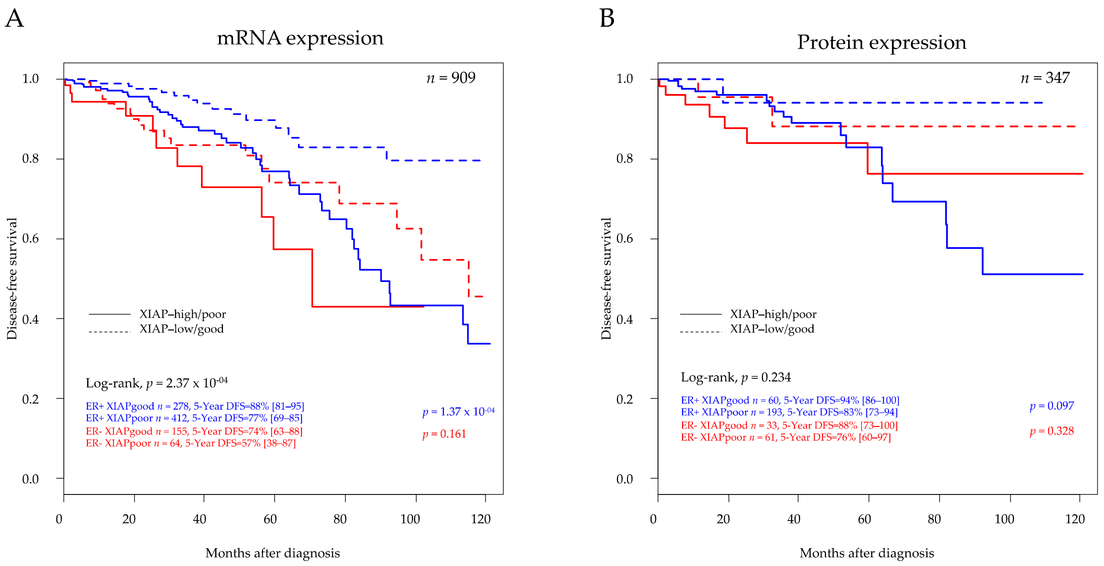

Figure S2. Disease-free survival according to XIAP expression in each ER status-based class. (**A**) Kaplan-Meier DFS curve in 909 patients *per* ER status and according to high and low *XIAP* mRNA expression. The XIAP-low and XIAP-high groups were defined by the Cox model prediction. (**B**) Kaplan-Meier DFS curve in 347 patients *per* ER status and according to high and low XIAP protein expression. The XIAP-low and XIAP-high groups were defined by the Cox model prediction.

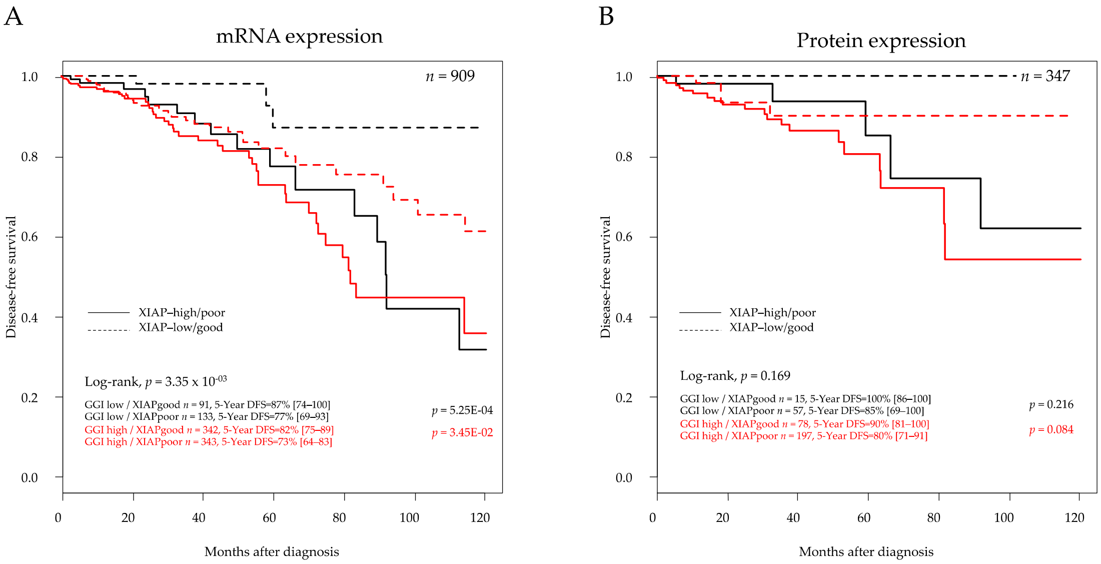

Figure S3. Disease-free survival according to XIAP expression in each grade-based class. (**A**) Kaplan-Meier DFS curve in 909 patients *per* grade-based class and according to high and low *XIAP* mRNA expression. The XIAP-low and XIAP-high groups were defined by the Cox model prediction. (**B**) Kaplan-Meier DFS curve in 347 patients *per* grade-based class and according to high and low XIAP protein expression. The XIAP-low and XIAP-high groups were defined by the Cox model prediction.

Table S1. List of breast cancer mRNA data sets included in the study.

Reference	Source of data	Access Date	N° of samples	Technological platform	N° of probe sets	N° of samples used in this study
Hess KR et al., J Clin Oncol 2006 [1]	MDA133	04/11/2007	133	Affymetrix U133A	22K	131
Bonnefoi et al., Lancet Oncol 2007 [2]	GEO: GSE6861, GSE4779	03/10/2010	161	Affymetrix X3P	61K	125
Iwamoto T et al., J Natl Cancer Inst 2011 [3]	GEO: GSE22093	06/14/2012	164	Affymetrix U133A	22K	100
Tabchy A et al., Clin Cancer Res 2010 [4]	GEO: GSE20271	06/14/2012	178	Affymetrix U133A	22K	178
Desmedt et al., J Clin Oncol 2011 [5]	GEO: GSE16446	01/23/2012	120	Affymetrix U133 Plus 2.0	54K	120
Hatzis C et al., JAMA 2011 [6]	GEO: GSE25066	01/03/2012	508	Affymetrix U133A	22K	504
Popovici V et al., Breast Cancer Res 2010 [7]	GEO: GSE20194	03/02/2012	278	Affymetrix U133A	22K	91
TCGA, Nature 2012 [8]	TCGA Data Portal - BRCA -	10/28/2013	1215	Illumina, RNAseq V2	20K	1092
TOTAL			2757			2341

Table S2. List of *XIAP* probe sets analyzed.

Technological platform		Probe set ID	Blastn / XIAP (3 transcripts)			Number of correspondin g data sets	Number of corresponding breast cancer samples*
			Query Cover	Identity	Specificity		
Affymetrix, U133+2.0 & U133A	Oligo-array, 25-mers	206536_S_AT	100%	100%	100%	6	1124
Affymetrix, X3P	Oligo-array, 25-mers	G1016687_3P_A_AT	100%	100%	100%	1	125

Table S3. XIAP protein expression and clinicopathological variables.

Variables		*n*	Global *n* (%)	XIAP protein mean (range)	*p*-value*
Age at diagnosis (year)					0.819
	≤50	106	106 (29%)	0.18 (–2.4–3.8)	
	>50	261	261 (71%)	0.10 (–3.9–2.9)	
Pathological type					3.35×10^{-4}
	IDC	315	315 (86%)	0.03 (–3.9–3.84)	
	ILC	29	29 (8%)	0.66 (–0.6–2.3)	
	other	23	23 (6%)	0.30 (–1.9–1.4)	
Pathological lymph node (pN)					4.13×10^{-2}
	negative	140	140 (47%)	0.01 (–3.6–2.3)	
	positive	158	158 (53%)	0.24 (–3.9–3.8)	
Pathological size (pT)					0.991
	pT1	80	80 (22%)	0.03 (–3.9–3.8)	
	pT2–3	286	286 (78%)	0.16 (–3.9–2.9)	
Genomic grade (GGI)					0.156
	low	75	75 (20%)	0.30 (–2.3–1.6)	
	high	291	291 (80%)	0.08 (–3.9–3.8)	
ER status**					1.74×10^{-2}
	negative	91	91 (25%)	–0.21 (–3.9–2.3)	
	positive	275	275 (75%)	0.20 (–3.9–3.8)	
PR status**					1.35×10^{-2}
	negative	142	142 (39%)	–0.12 (–3.9–2.3)	
	positive	224	224 (61%)	0.25 (–2.6–3.8)	
HER2 status**					0.373
	negative	297	297 (81%)	0.16 (–3.9–3.8)	
	positive	69	69 (19%)	0.02 (–2.4–2.3)	
Molecular subtype mRNA status					0.076
	HR+/HER2-	237	237 (65%)	0.20 (–3.9–3.8)	
	HER2+	69	69 (19%)	0.02 (–2.4–2.3)	
	TN	61	61 (17%)	–0.19 (–3.9–2.3)	
PAM50 subtypes					3.12×10^{-3}
	basal	88	88 (24%)	–0.30 (–3.9–2.3)	
	HER2	63	63 (17%)	0.18 (–2.4–2.3)	
	luminal A	82	82 (22%)	0.25 (–3.9–1.6)	
	luminal B	108	108 (30%)	0.21 (–2.3–3.8)	
	normal-like	25	25 (7%)	0.38 (–2.2–2.3)	
DFS event	no	315	315 (91%)	0.12 (–3.9–3.84)	0.250
	yes	32	32 (9%)	0.21 (–1.46–2.19)	
5-year DFS [95% CI]		347	84% [78–91]		

GGI, genomic grade index; IDC, invasive ductal carcinoma; ILC, invasive lobular carcinoma; * Student's t-test or one-way ANOVA; **, mRNA status.

Table S4. Univariate and multivariate analyses for DFS in the "RNA population" by using XIAP expression as discrete value.

Variables	Univariate *n*	Univariate Hazard ratio [95%CI]	Univariate *p*-value	Multivariate *n*	Multivariate Hazard ratio [95CI]	Multivariate *p*-value
Age at diagnosis, >50 vs. ≤50	909	1.24 [0.81–1.88]	0.323			
Genomic grade (GGI), high vs. low	909	1.30 [0.81–2.07]	0.275			
Pathological lymph node, 1 vs. 0	776	2.05 [1.32–3.18]	1.40×10^{-3}	776	1.96 [1.24–3.1]	3.70×10^{-3}
Pathological size, pT2–3 vs. pT1	908	1.15 [0.74–1.79]	0.536			
Pathological type, lobular vs. ductal	909	0.54 [0.28–1.04]	0.182			
other vs. ductal		0.99 [0.55–1.81]				
Mol. subtype, HER2+ vs. HR+/HER2-	909	2.18 [1.31–3.63]	7.72×10^{-4}	776	1.07 [0.53–2.14]	0.857
Mol. subtype, TN vs. HR+/HER2-		2.13 [1.32–3.43]		776	1.78 [1.04–3.05]	3.48×10^{-2}

Amsterdam 70-gene, Poor vs. Good	909	2.46 [1.31–4.60]	4.89×10^{-3}	776	2.01 [1.04–3.88]	3.70×10^{-2}
OncotypeDX, High vs. Low	909	1.60 [0.98–2.60]	0.168			
Intermediate vs. Low		1.33 [0.70–2.51]				
XIAP high *vs.* Low (discrete value)	909	2.01 [1.35–3]	5.75×10^{-4}	776	2.17 [1.39–3.4]	7.10×10^{-4}

Table S5. Univariate and multivariate analyses for DFS in the "RPPA population".

Variables	*n*	Univariate HR [95%CI]	*p*-value
Age at diagnosis, >50 *vs.* ≤50 years	347	1.54 [0.71–3.35]	0.272
Genomic grade (GGI), high *vs.* low	346	1.34 [0.57–3.13]	0.499
Pathological lymph node, pos. *vs.* neg.	288	1.09 [0.50–2.34]	0.832
Pathological size, pT2–3 *vs.* pT1	347	1.12 [0.50–2.50]	0.776
Pathological type, ILC *vs.* IDC	347	1.42 [0.42–4.79]	0.491
other *vs.* IDC		1.70 [0.67–4.29]	
Mol. subtype, HER2+ *vs.* HR+/HER2-	346	2.33 [0.99–5.50]	0.151
Mol. subtype, TN *vs.* HR+/HER2-		1.15 [0.43–3.13]	
Amsterdam 70-gene risk, high *vs.* low	346	2.12 [0.74–6.09]	0.162
Recurrence Score risk, high *vs.* low	346	1.20 [0.53–2.71]	0.436
intermediate *vs.* low		0.54 [0.14–2.06]	
*XIAP*continuous expression	347	1.50 [1.02–2.22]	4.15×10^{-2}

GGI, genomic grade index; IDC, invasive ductal carcinoma; ILC, invasive lobular carcinoma; HR, Hazard ratio.

Table S6. Univariate and multivariate analyses for pCR to neoadjuvant chemotherapy by using XIAP expression as discrete value.

pCR	Univariate *n*	Univariate Odds-ratio [CI95]	Univariate *p*-value	Multivariate *n*	Multivariate Odds-ratio [CI95]	Multivariate *p*-value
Age at diagnosis (years), >50 vs. ≤50	1202	0.86 [0.68–1.10]	0.262			
Genomic Grade Index (GGI), high vs. low	1203	2.10 [1.70–2.80]	7.65×10^{-7}	1203	1.6 [1.2–2.1]	3.45×10^{-3}
Pathological lymph node status (pN), positive vs. negative	253	0.83 [0.53–1.30]	0.508			
Pathological tumor size (pT), pT2–3 vs. pT1	299	1.10 [0.58–2.00]	0.871			
Pathological type, ILC vs. IDC	510	1.60 [0.63–4.30]	0.397			
Pathological type, other vs. IDC	510	0.75 [0.46–1.20]	0.314			
Molecular subtype, HER2+ vs. HR+/HER2-	1203	3.80 [2.70–5.40]	1.12×10^{-10}	1203	3.5 [2.5–4.9]	4.03×10^{-9}
Molecular subtype, TN vs. HR+/HER2-	1203	3.60 [2.80–4.70]	2.22×10^{-15}	1203	3.1 [2.4–4.1]	8.77×10^{-12}
XIAP, high *vs.*Low (discrete value)	1203	0.71 [0.57–0.9]	1.53×10^{-2}	1203	0.79 [0.62–1]	9.32×10^{-2}

References

1 Hess, K.R.; Anderson, K.; Symmans, W.F.; Valero, V.; Ibrahim, N.; Mejia, J.A.; Booser, D.; Theriault, R.L.; Buzdar, A.U.; Dempsey, P.J.; et al. Pharmacogenomic Predictor of Sensitivity to Preoperative Chemotherapy with Paclitaxel and Fluorouracil, Doxorubicin, and Cyclophosphamide in *Breast Cancer. J. Clin. Oncol.* **2006**, *24*, 4236–4244, doi:10.1200/JCO.2006.05.6861.

2 Bonnefoi, H.; Potti, A.; Delorenzi, M.; Mauriac, L.; Campone, M.; Tubiana-Hulin, M.; Petit, T.; Rouanet, P.; Jassem, J.; Blot, E.; et al. Validation of Gene Signatures That Predict the Response of Breast Cancer to Neoadjuvant Chemotherapy: A Substudy of the EORTC 10994/BIG 00-01 *Clinical Trial. Lancet Oncol.* **2007**, *8*, 1071–1078, doi:10.1016/S1470-2045(07)70345-5.

3 Iwamoto, T.; Bianchini, G.; Booser, D.; Qi, Y.; Coutant, C.; Shiang, C.Y.-H.; Santarpia, L.; Matsuoka, J.; Hortobagyi, G.N.; Symmans, W.F.; et al. Gene Pathways Associated with Prognosis and Chemotherapy Sensitivity in Molecular Subtypes of *Breast Cancer. J. Natl. Cancer Inst.* **2011**, *103*, 264–272, doi:10.1093/jnci/djq524.

4 Tabchy, A.; Valero, V.; Vidaurre, T.; Lluch, A.; Gomez, H.; Martin, M.; Qi, Y.; Barajas-Figueroa, L.J.; Souchon, E.; Coutant, C.; et al. Evaluation of a 30-Gene Paclitaxel, Fluorouracil, Doxorubicin, and Cyclophosphamide Chemotherapy Response Predictor in a Multicenter Randomized Trial in *Breast Cancer. Clin. Cancer Res.* **2010**, *16*, 5351–5361, doi:10.1158/1078-0432.CCR-10-1265.

5 Desmedt, C.; Di Leo, A.; de Azambuja, E.; Larsimont, D.; Haibe-Kains, B.; Selleslags, J.; Delaloge, S.; Duhem, C.; Kains, J.-P.; Carly, B.; et al. Multifactorial Approach to Predicting Resistance to Anthracyclines. *J. Clin. Oncol.* **2011**, *29*, 1578–1586, doi:10.1200/JCO.2010.31.2231.

6 Hatzis, C.; Pusztai, L.; Valero, V.; Booser, D.J.; Esserman, L.; Lluch, A.; Vidaurre, T.; Holmes, F.; Souchon, E.; Wang, H.; et al. A Genomic Predictor of Response and Survival Following Taxane-Anthracycline Chemotherapy for Invasive Breast Cancer. *JAMA* **2011**, *305*, 1873–1881, doi:10.1001/jama.2011.593.

7 Popovici, V.; Chen, W.; Gallas, B.G.; Hatzis, C.; Shi, W.; Samuelson, F.W.; Nikolsky, Y.; Tsyganova, M.; Ishkin, A.; Nikolskaya, T.; et al. Effect of Training-Sample Size and Classification Difficulty on the Accuracy of Genomic Predictors. *Breast Cancer Res.* **2010**, *12*, R5, doi:10.1186/bcr2468.

8 Koboldt, D.C.; Fulton, R.S.; McLellan, M.D.; Schmidt, H.; Kalicki-Veizer, J.; McMichael, J.F.; Fulton, L.L.; Dooling, D.J.; Ding, L.; Mardis, E.R.; et al. Comprehensive molecular portraits of human breast tumours. Nature 2012, 490, 61–70, doi:10.1038/nature11412.
